# Anatomical characterization of sternoclavicular joint and correlation of arthroscopic portals and structures at risk: a cadaveric study on Colombian specimen

**DOI:** 10.1016/j.xrrt.2025.100650

**Published:** 2025-12-24

**Authors:** Fabio Alfonso Suarez Romero, Deisy Consuelo Celeita Medina, María Camila Ruiz Cardenas, Andrea Juliana Hernández Caicedo, Federico Suarez

**Affiliations:** a Department of Hand and Upper Extremity Surgery, Central Military Hospital, Bogotá, Colombia; b Nueva Granada Military University, Central Military Hospital, Bogotá, Colombia; c Nueva Granada Military University, Bogotá, Colombia

**Keywords:** Sternoclavicular joint, Arthroscopy, Mediastinum, Posterior capsule, Cadaveric study, Neurovascular safety

## Abstract

**Background:**

The sternoclavicular joint (SCJ) is a synovial saddle joint and the only articulation between the axial skeleton and the upper limb. Although SCJ pathology is rare, it may include degenerative, autoimmune, infectious, tumoral, or traumatic conditions. Arthroscopic approaches to this region carry a risk of damaging vital mediastinal structures. This study aims to anatomically characterize the SCJ and surrounding neurovascular structures, emphasizing the posterior capsule as a critical safety barrier during arthroscopy.

**Methods:**

Ten cadaveric specimens were bilaterally dissected. Arthroscopic portals were first marked and used to identify the posterior capsule. Open dissections were performed to measure the distances between the portals and nearby mediastinal structures using digital calipers. Data were recorded in Excel 2024 (Microsoft Corp., Redmond, WA, USA) and REDCap (Vanderbilt University, Nashville, TN, USA) and analyzed with SPSS version 28 (IBM Corp., Armonk, NY, USA).

**Results:**

The posterior capsule consistently acted as a protective boundary between the joint and mediastinal structures. The average distances from the superomedial (SM) and inferolateral (IL) portals to the posterior capsule were 26.3 mm and 26.5 mm, respectively. From the posterior capsule, the average distances to key mediastinal structures were: common carotid artery: 42.5 mm (SM), 44.1 mm (IL), brachiocephalic trunk: 40.5 mm (SM), 43.5 mm (IL), innominate vein: 37.8 mm (SM), 39.7 mm (IL), and vagus nerve: 45.4 mm (SM), 46.5 mm (IL).

**Discussion and/or Conclusion:**

The posterior capsule provides a reliable anatomical safety margin between the SCJ and vital mediastinal structures. During SCJ arthroscopy, referencing the anterior sternoclavicular ligament and maintaining a minimum 5.1 cm safety margin posteriorly can minimize the risk of catastrophic injury. These findings support safer surgical planning and highlight the importance of precise portal placement.

The sternoclavicular joint (SCJ) can be affected by degenerative, traumatic, and immunological pathologies, which can limit shoulder mobility and disturb quality of life. There are limited reports of these pathologies, and they are most often managed in a conservative manner due to the limited knowledge of the anatomy of this joint and its proximity to mediastinal structures, which are at risk and can have fatal outcomes.^1^, ^2^, ^3^ The main indications for performing SCJ arthroscopy include degenerative arthritis, synovitis, the presence of loose bodies, and intra-articular impingement.^4^, ^5^, ^6^

With proper technique and knowledge of the joint's anatomy, the arthroscopy approach has evolved into a safe, reproducible, and minimally invasive procedure allowing resection of the intra-articular disc or the medial third of the clavicle in degenerative cases, removal of loose bodies, and débridement in cases of infection.^7^

According to descriptive cadaveric studies, the joint has multiple stabilizers such as the posterior sternoclavicular ligament, the anterior sternoclavicular ligament, the costoclavicular ligament (being the largest), the interclavicular ligament, and the intra-articular disc. Bony landmarks include the crest of the pectoralis major and the insertion of the sternohyoid and sternothyroid muscles immediately posteriorly, between which an avascular zone "safe zone" is described.^5^

Regarding the mediastinal structures, the left brachiocephalic vein is the closest to the SCJ, followed by the right, then the right common carotid artery, aortic arch, trachea, superior vena cava, pleura, and left common carotid artery. However, these findings are based on tomographic studies rather than anatomical dissections.^1^

In this study, we aim to describe the anatomical correlation of the arthroscopic portals of the SCJ in cadaveric specimens and its proximity to neurovascular structures at risk of injury. Because there is no literature on this topic in Colombia and information is rare worldwide, this study attempts to address a significant gap in our understanding of anatomy and surgical technique.

## Materials and methods

This descriptive anatomical study was carried out in the Latin American Center for Research and Training in Minimally Invasive Surgery in Bogota, Colombia. With a sample of 6 thawed fresh-frozen cadavers who underwent bilateral procedures, and a total of 20 dissections were performed.

The only inclusion criterion was the absence of previous shoulder pathologies or deformities. All specimens lacked history of traumatic shoulder pathologies, previous dislocations, or obvious morphological deformities. The age and sex of the cadavers were not considered.

**Figure 1:**
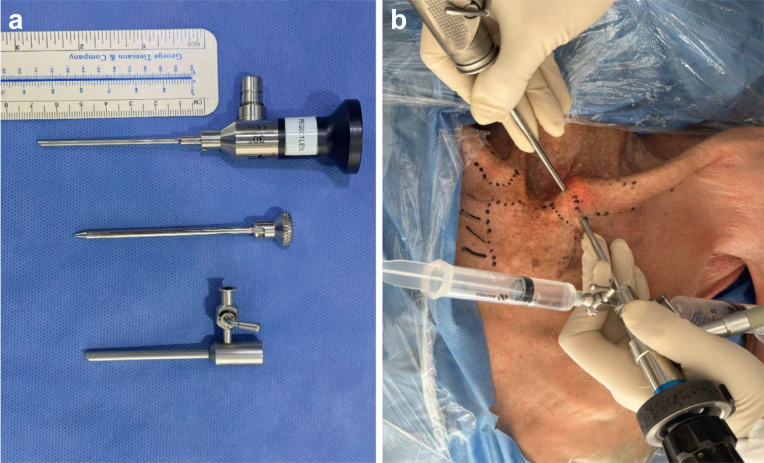
(**a**) Arthroscopic lenses. (**b**) Arthroscopic portals.
b) The articular surface, meniscus, and posterior capsule were visualized.
c) The distance from the superior and inferior arthroscopic portals to the posterior capsule was measured from the SM and IL arthroscopic portals to the main anatomical structures at risk (Fig. 1, *b*).

The dissection process was carried out in 2 phases as follows:

First phase:

a) A complete diagnostic arthroscopy of the SCJ was performed using a 5.7 mm diameter by 60 mm long lens (Fig. 1, *a*) creating a superomedial (SM) and inferolateral (IL) portals.

**Figure 2:**
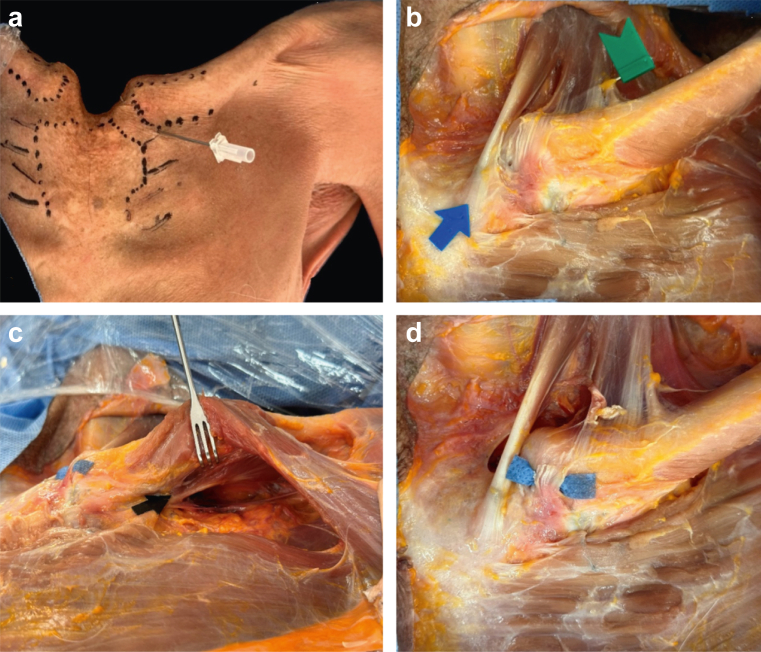
(**a**) Anatomical landmarks for sternoclavicular joint. (**b**) *Green flag*: clavicular portion of the sternocleidomastoid muscle; *blue arrow*: sternal portion of sternocleidomastoid muscle. (**c**) Subclavius muscle. (**d**) Anterior sternoclavicular ligament.
b) The sternoclavicular structures were exposed, including the pectoralis major, subclavius, sternocleidomastoid, sternohyoid, and sternothyroid muscles (Fig. 2, *b* and *c*).
c) Identification of anterior, superior, costoclavicular, and sternoclavicular ligaments (Fig. 2, *d*).
d) A rectangular osteotomy was created using a saw that included the sternum, middle third of both clavicles, and first 4 ribs (Fig. 3, *a*).

Second phase:

a) A Y-incision was made over the sternum (Fig. 2, *a*) with careful dissection.

**Figure 3:**
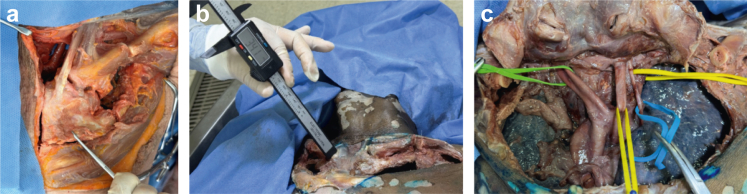
(**a**) Osteotomy of sternoclavicular joint. (**b**) Measurements to main vascular structures. (**c**) Mediastinal vascular structures. *Green*: brachiocephalic trunk; *yellow*: common carotid artery; and *blue*: vagus nerve.
e) Distances were measured from the SM and IL portals to the great vessels and structures at risk (Fig. 3, *b*):
a) Brachiocephalic trunk
b) Common carotid artery
c) Innominate vein
d) Vagus nerve
f) The same protocol was repeated contralaterally (Fig. 3, *c*).

### Statistical analysis

Quantitative variables were collected in an Excel matrix and in REDCap, enabling an univariate statistical analysis and to determine the characteristics of the study population. For continuous variables, descriptive measures were summarized reporting the mean and standard deviation in those values that were normally distributed. Since bilateral dissections were performed on each cadaver, a bivariate analysis by laterality was carried out for each variable applying the Wilcoxon test. Finally, a general correlation between all variables was performed using the Pearson correlation coefficient to evaluate the linear relationship. This protocol was approved by the ethics committee of the Central Military Hospital of Bogotá before starting data collection.

## Results

Ten cadaveric specimens were collected, performing a total of 20 dissections. Of these specimens 90% were male, with a median age of 66 years (interquartile range 55-70 years). Arthroscopic approach to the SCJ was performed using a 5.7 mm lens to visualize the articular surface, the meniscus, and the posterior capsule, which was constant in all views. The latter was found as a safe limit for the mediastinum.

In addition, the anterior sternoclavicular ligament was established as a reference point for the correct positioning of portals. The average distance from the SM and IL to the posterior capsule was 26.3 mm an 26.5 mm, respectively.

During the cadaveric dissection, the distance from the arthroscopic portals to the main neurovascular structures were measured, including the common carotid artery, the brachiocephalic trunk, the innominate vein, and the ipsilateral vagus nerve (Table I).

**Table I:** Summary of distances from portals to neurovascular structures.

Ref-cadaver	Sex	Age	Side	Distance from portal to neurovascular structure in millimeters									
				SM-Pc	SM-Cc	SM-Bt	SM-Vn	SM-In	IL-Pc	IL-Cc	IL-Bt	IL-Vn	IL-In
0250	M	46	L	28	20	30	40	29	30	22	33	42	30
			R	30	25	32	42	28	32	24	34	44	31
0998	M	46	L	30	38	34	37	32	30	35	38	34	32
			R	33	39	35	37	30	32	36	40	35	31
0064	F	64	L	25	43	37	37	32	26	44	45	37	31
			R	25	40	34	36	32	24	44	35	36	33
0889	M	70	L	25	48	40	42	41	26	42	54	48	46
			R	29	46	43	46	44	25	49	50	49	47
4439	M	72	L	26	41	41	47	37	26	51	46	55	40
			R	25	45	35	50	33	22	51	39	56	45
4455	M	74	L	20	38	37	40	37	21	40	32	42	30
			R	24	39	40	47	38	20	44	43	46	41
0345	M	69	L	25	51	52	58	44	28	55	51	55	45
			R	29	50	52	55	48	25	52	47	54	45
2324	M	68	L	23	50	53	58	45	27	47	53	58	46
			R	24	53	58	60	41	33	57	58	60	45
4945	M	55	L	26	47	38	45	42	25	49	42	44	41
			R	25	48	37	46	40	26	50	40	45	42
5658	M	57	L	28	44	42	48	43	27	46	42	45	43
			R	27	46	40	47	41	26	44	43	46	44

*M*, male; *F*, female; *R*, right; *L*, left; *Pc*, posterior capsule; *SM*, superomedial; *Cc,* common carotid; *Bt,* brachiocephalic; *Vn*, vagus nerve; *In*, innominate vein; *IL*, inferolateral portal.

The average distance from the SM portal to the common carotid artery was 42.5 mm and 44.1 mm for the IL portal, with no statistically significant differences based on laterality. Similarly, the average distance to the brachiocephalic trunk was 40.5 mm from SM and 43.5 mm from IL. The distance to the innominate vein was 37.8 mm from SM and 39.7 mm from IL, this being the closest structure to the arthroscopic portals. Finally, the average distance to the ipsilateral vagus nerve was 45.4 mm from SM and 46.5 mm from IL portal.

**Figure 4:**
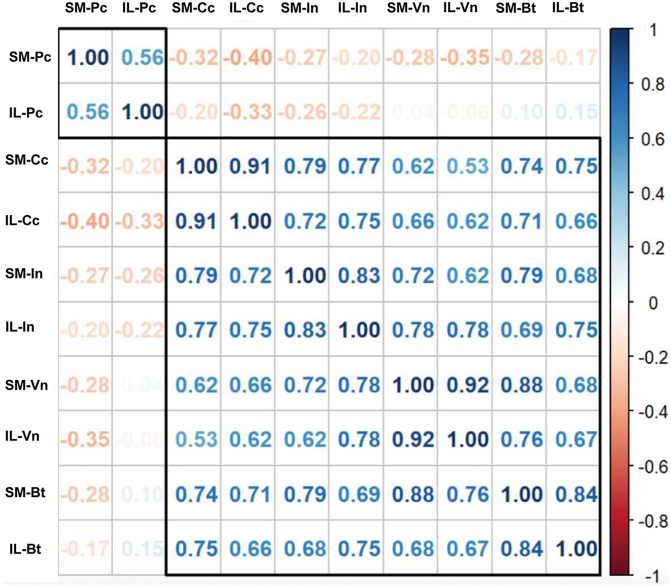
Correlation matrix between the distances measured from the SM and IL arthroscopic portals to key anatomical structures. *SM*, superomedial; *IL*, inferolateral; *Pc*, posterior capsule; *Cc*, common carotid; *Bt*, brachiocephalic trunk; *Vn*, vagus nerve; *In*, innominate vein.

A Pearson correlation analysis was performed between the variables (Fig. 4). Correlation coefficients ranged from −0.40 to 0.92, reflecting a wide variety of linear relationships. Strong positive correlation was observed between arthroscopic portals and the large neurovascular structures, suggesting a significant association between them. On the other hand, a negative correlation was found between arthroscopic portals and the posterior capsule. These results are congruent with anatomical observations indicating that an increase in depth from the portals to the posterior capsule decreases the distance to the neurovascular structures.

## Discussion

The SCJ is a saddle-shaped synovial joint being the only skeletal connection between the axial skeleton and the upper limb. Pathologies affecting this joint are rare but often debilitating and underdiagnosed due to limited anatomical knowledge.

In the literature, corticosteroid injections are commonly used as conservative therapy. Open surgical procedures are used when pain is persistent. These procedures, however, include the risk of joint instability, scarring, and injury to the underlying mediastinal structures. Over the past five years, advancements in arthroscopy technique have lowered the possibility of damaging critical structures and provide a safer, less-invasive technique.^7^

Regarding the anatomical characterization of the SCJ, the union between the medial end of the clavicle, the clavicular notch on the sternum, and the first rib cartilage contributes to stability.^3^ The costoclavicular ligament, the anterior and posterior sternoclavicular ligaments, and the interclavicular ligament all contribute to its stability. These findings were consistent with the 20 anatomical dissections performed, with all components identified and the anterior sternoclavicular ligament established as the portal limit.

Arthroscopic portals of the SCJ are described as SM and IL. The former located medial to the anterior sternoclavicular ligament, whereas the IL portal is established at the level of the joint line, just lateral to the anteroinferior border of the clavicle.^2^ Since the posterior capsule's thick wall keeps the lens from entering the mediastinum, it was determined to be a safety limit to prevent injury to mediastinal structures. The average distance from SM and IL portal to the posterior capsule was 26.3 and 26.5 mm, respectively. This indicates that, in contrast to literature references, the safe depth of the SCJ in the Colombian population is 5.1 cm in all directions, beyond which the posterior capsule is breached and the danger of possibly fatal injury increases.

The innominate vein is the closest neurovascular structure at risk, with no statistical difference between the right and left side, averaging 3.6 cm from the SM portal and 3.4 cm from the IL. Followed by the brachiocephalic trunk with an average of 40.5 mm and 43.5 mm, respectively. In third place we found the common carotid artery, with an average of 7.25 cm from SM and 7.41 cm IL. Lastly, the vagus nerve is the most distant structure.

These data suggest that a vascular structure is more likely to be injured since it is close to the posterior capsule than a neurological structure. Furthermore, it became apparent that the greater the distance between the portals and the posterior capsule, the shorter the distance to the great vessels, confirming the posterior capsule as a critical safety margin.

In contrast to our investigation, the literature states that the aortic arch, common carotid artery, and innominate vein are the closest structures.^1^ However, measurements range between 1 and 6 cm, and in our study, lower measurement values were found, probably due to Colombian cadaver size.

### Limitations

Our study has limitations related to sample selection and size given the availability of cadavers at Latin American Center for Research and Training in Minimally Invasive Surgery institute. We were unable to consider the sex or age of specimens.

## Conclusion

Anatomical knowledge of the SCJ is essential when considering surgical approach. Our study demonstrated that the innominate vein, the common carotid artery, and the brachiocephalic trunk are the nearest vascular structures, followed by the vagus nerve. If an arthroscopic approach is chosen, the anterior sternoclavicular ligament should be used as a reference point for portal creation, and the posterior capsule as a safety margin, ensuring a safe depth of 5.1 cm for joint manipulation with arthroscopic instruments to reduce the risk of mediastinal structure injury.

## Disclaimers

Funding: No funding was disclosed by the authors.

Conflicts of interest: The authors, their immediate families, and any research foundation with which they are affiliated have not received any financial payments or other benefits from any commercial entity related to the subject of this article.
